# Pneumonia among Under-Five Children in Northwest Ethiopia: Prevalence and Predictors—A Community-Based Cross-Sectional Study

**DOI:** 10.1155/2020/3464907

**Published:** 2020-04-29

**Authors:** Zewudu Andualem, Tsegaye Adane, Abiye Tigabu, Walelign Worku Yallew, Sintayehu Daba Wami, Henok Dagne, Jember Azanaw, Gebisa Guyasa, Zelalem Nigussie Azene, Mastewal Endalew

**Affiliations:** ^1^Department of Environmental and Occupational Health and Safety, Institute of Public Health College of Medicine and Health Sciences, University of Gondar, Gondar, Ethiopia; ^2^Department of Medical Microbiology, School of Biomedical and Laboratory Sciences, College of Medicine and Health Sciences, University of Gondar, Gondar, Ethiopia; ^3^Department of Women and Family Health, School of Midwifery, College of Medicine and Health Sciences, University of Gondar, Gondar, Ethiopia

## Abstract

**Background:**

Acute respiratory infections in particular pneumonia constitutes the leading cause of morbidity and mortality among children under five years of age throughout the world. In Ethiopia, pneumonia continues to be the major childhood problem and killer, particularly in the study area. However, evidence dealing with the problem is still unavailable. The current study is aimed at determining the magnitude and risk factors of childhood pneumonia in Gondar City.

**Methods:**

A community-based cross-sectional study was employed in five randomly selected clusters/subcities of Gondar City. A total of 792 child-mother/caregiver pairs in the selected subcities/clusters were included. A pretested and validated questionnaire was used by trained supervisors through house-to-house visits to collect the data. Binary logistic regression (bivariable and multivariable) was employed. An adjusted odds ratio with 95% confidence interval was used to declare statistically significant variables on the basis of *p* < 0.05 in the multivariable logistic regression model.

**Results:**

The prevalence of pneumonia among under-five children in the current study was found to be 12% with 95% CI: 10% to 14.4%. The presence of unpaved road within 100 m of the house (AOR = 2.27, 95% CI: 1.41-3.66), living within 100 m of heavy traffic (AOR = 1.94, 95% CI: 1.19-3.16), the habit of not opening doors while cooking (AOR = 1.62, 95% CI: 1.01-2.62), the presence of cockroach infestation (AOR = 1.98, 95% CI: 1.25-3.14), and new carpet in the house (AOR = 1.75, 95% CI: 1.01-3.03) were statistically significant variables associated with childhood pneumonia.

**Conclusions:**

This study indicated that the prevalence of childhood pneumonia is still high. As such, enhancing strategies that would address unpaved roads within 100 m of the house, living within 100 m of heavy traffic, the habit of not opening doors while cooking, cockroach infestation, and new carpet in the house to reduce the burden of childhood pneumonia needs to be advocated.

## 1. Introduction

Pneumonia is one form of an acute lower respiratory infection that affects the lungs. It is the single most important infectious cause of death in children globally. Annually, an estimated 921,000 children younger than 5 years die of pneumonia in 2015. More than 95% of these deaths occur in low- and middle-income countries (LMIC) predominantly South Asia and sub-Saharan Africa [1–3].

Pneumonia is the leading cause of morbidity and mortality among children below five years of age in Ethiopia, with an approximately 3,370,000 children experiencing pneumonia every year that attributes to 20% of all causes of deaths and killing more than 40,000 under five-children annually, making it the number one cause of death during the postnatal period as well [4, 5]. A study carried out in Dibrugarh town, India, demonstrated that 16.34% of under-five children suffered from pneumonia [6]. According to a study conducted in the Ekiti State, Nigeria, the overall magnitude of Acute Respiratory Infection (ARI) among children below five years was 64.9% [7]. The very recent 2016 Ethiopian Demographic Health Survey (EDHS) revealed that the prevalence of ARI in Ethiopia is 18% [8]. Other local studies conducted at different regions of Ethiopia at different times reported that the overall two-week prevalence of pneumonia among under-five children was 16.1% [9] and 33.5% [10].

Previous evidences identified that a range of factors were related to the occurrence of pneumonia among under-five children. These factors include low socioeconomic status, low educational level of mothers, absence of a separate kitchen, absence of windows in the kitchen, breastfeeding less than one year and children at age range of 2-12 months, partial immunization, poor practices related to child feeding and hand hygiene, poor knowledge related to signs and symptoms of pneumonia among mothers, overcrowding, indoor air pollution, charcoal use for cooking, carrying the child on back during cooking, cockroach infestation, new furniture, and redecoration [10–17].

Though pneumonia is the number one fatal common cause among under-five children, there is no sufficient evidence about the prevalence of pneumonia and the associated factors in Ethiopia, in the study area in particular and in this segment of population. Thus, this study was aimed at assessing the prevalence of pneumonia and associated factors among under-five children in Gondar City, Northwest Ethiopia.

## 2. Methods

### 2.1. Study Setup, Design, and Period

A community-based cross-sectional study was conducted from February 15, 2018, to June 20, 2019, in Gondar City, Northwest Ethiopia. The city is located in Central Gondar Zone, Amhara Regional State of Ethiopia, and is far 750 km northwest of Addis Ababa, capital of Ethiopia, and, about 180 kilometers from Bahir Dar town, the capital of the Amhara regional state. The city has an altitude of 12°36′N 37°28′E and longitude of 12.60N 37.467′E with an elevation of 2133 meters above sea level and is divided into 12 administrative areas (subcities) which consist of 21 kebeles (the smallest administrative unit in Ethiopia). Gondar is among one of the ancient and largely populated cities in the country. The city has an estimated total population of 324,000 with a total of 23,929 under-five children. There are one public hospital and eight health centers providing child and other health services to the population.

### 2.2. Sample Size Calculation and Sampling Procedure

The single population proportion formula was used to calculate the sample size considering the following assumptions: *p* = 50% proportion of children with respiratory symptoms (there is no previous study in the study area), 95% confidence interval, a 5% margin of error (*d*), and design effect 2:

(1) $$\begin{array}{c} n=\frac{{\left(\text{Za}/2\right)}^{2}*P\left(1‐P\right)}{d^{2}},\\ n=\frac{{\left(1.96\right)}^{2}*0.5\left(1-0.5\right)}{0.05^{2}}=384. \end{array}$$

By taking 5% of the nonresponse rate, then the total ample size was 806.

The 12 administrative areas of the cities were used for multistage sampling with an assumption of being heterogeneous. 50% of the total subcities were selected randomly from the 12 administrative areas, and all eligible study participants present in the selected subcities were included in the study.

### 2.3. Data Collection and Analysis

Data were collected using a pretested and semistructured questionnaire through face-to-face interview at the participant's home. The questionnaire was first prepared in English and then translated to Amharic (local language) and back to English to maintain consistency of the tool. In the data collection process, six diploma nurses and one BSc nurse were involved. A two-days training was provided for data collectors and supervisors.

Data were first checked manually for completeness and then coded and entered into Epi Info version 7.1.2.0. Then, the data were exported to Statistical Package for the Social Sciences (SPSS) version 20.00 for data checking, cleaning, and analysis. Descriptive statistics were performed to describe the study population in relation to dependent and independent variables. Binary logistic regression (bivariable and multivariable logistic regression) was used to identify statistically significant independent variables, and independent variables having a *p* value less than 0.2 in the bivariable analysis were entered into multivariable logistic regression for further analysis. *p* value of 0.05 was considered to declare the level of significance.

## 3. Results

### 3.1. Sociodemographic Profile of Study Participants

A total of 806 mothers/caregivers and children pair were selected for this study. Of these, 792 participants were enrolled with a response rate of 98.3%. The mean age of the children was 2.51 years with SD ± 1.21 years and that of mothers was 29.1 years with SD ± 5.9 years. About 610 (77.0%) of mothers/guardians were Orthodox Christian followers. In terms of educational status, 253 (31.9%) of them had attended secondary education whereas 135 (17.0%) of them had no formal education. Regarding occupational status, 541 (68.3%) of the respondents' occupation was a housewife (Table 1).

### 3.2. Prevalence of Pneumonia among Under-Five Children

The prevalence of pneumonia among under-five children in the current study was 12% with 95% CI (10% to 14.4%).

Child exposure to pets, the presence of unpaved road within 100 m of the house, living within 100 m of heavy traffic, the habit of not opening doors while cooking, the presence of damp stain, cockroach infestation, new carpet in the house, and maternal wheezing and coughing symptoms were candidates for multivariable binary logistic regression at a cut-off point of *p* < 0.2. Only the presence of unpaved road within 100 m of the house, living within 100 m of heavy traffic, the habit of not opening doors while cooking, cockroach infestation, and new carpet in the house were significantly associated with pneumonia among under-five children in the final multivariable binary logistic regression analysis (*p* < 0.05).

Under-five children living within 100 meters from unpaved new road and heavy traffic road were 2.27 times (AOR: 2.27, 95% CI: 1.41, 3.66) and 1.94 times (AOR: 1.94, 95% CI: 1.19, 3.16) more likely to be at risk of getting pneumonia as compared with those who live far from unpaved and heavy traffic roads, respectively.

Children under the age of five who live in households who do not have a habit of opening doors and allowing ventilation during cooking were 1.62 times more likely at higher risk of developing pneumonia (AOR: 1.62, 95% CI: 1.01, 2.62). The odds of pneumonia among under-five children whose households have new carpet in the previous six months prior to data collection were 1.75 times higher as compared to those children who live in households with no new carpet (AOR: 1.75, 95% CI: 1.01, 3.03).

Cockroach infestation was another risk factor for pneumonia among under-fives in the current study. Children who live in cockroach-infested houses were 1.98 times more likely to be at higher risk of pneumonia (AOR: 1.98, 95% CI: 1.25, 3.14) (Table 2).

## 4. Discussion

The prevalence of pneumonia among under-five children in the current study was 12% with 95% CI (10% to 14.4%). This result was lower than that of a study conducted in Arisi zone, Ethiopia (17.7%) [18], Sidama zone, Ethiopia (33.3%) [10], Este town and the surrounding rural Kebeles, Northwest Ethiopia (16.1%) [9], Dibrugarh town, India (16.34%) [6], and West Nusa Tenggara, Indonesia (33.3%) [19]. The possible reason for the difference in the prevalence of pneumonia might include the time of data collection, assessment method used, and difference in the level of advancement as well as the aggregation of risk factors. However, the current pneumonia prevalence was in line with a report from Brazzaville, Republic of Congo (12.3%) [20].

The presence of unpaved road within 100 m of the house, living within 100 m of heavy traffic, the habit of not opening doors while cooking, presence of damp stain, cockroach infestation, and new carpet in the house were risk factors of pneumonia among under-five children in the current study.

The presence of unpaved road within 100 m of the house and living within 100 m of heavy traffic are risk factors for pneumonia in the sense of exposure to outdoor air pollutants. There is a well-established evidence that exposure to air pollution results in a higher risk of pneumonia [21–26].

The habit of not opening doors while cooking is a risk factor to childhood pneumonia in the current study. As most of the houses do not have a sufficient number of functional windows in this study area, opening doors while cooking is the most feasible way of ventilation. Poor ventilation was identified as a risk factor for childhood pneumonia in previous studies [27–30], too. Inadequate ventilation increases the house's internal moisture and provides a nurturing environment for mites, respiratory viruses, and molds that play role in respiratory disease pathogenesis [31]. Ventilation can promote microbe clearance from the household atmosphere by improving natural air circulation, thus directly decreasing the risk of exposure to respiratory pathogens for under-five children [32]. In the current study, the presence of new carpet in houses was a risk factor for childhood pneumonia. The presence of new furniture such as carpet has been associated with childhood respiratory illnesses in earlier studies as well [16, 33, 34]. Carpets are likely to harbor house dust and thereby pneumonia-causing pathogens [35]. In particular, synthetic fiber carpet had the greatest association with pneumonia as indicated in a study [34].

Finally, under-five children who live in houses infested by cockroaches were 1.98 times more likely to be at risk of developing pneumonia. A study in the same site indicated that cockroaches are a source of high bacterial pathogens with multidrug-resistant strains [36]. A similar finding that supports this result was reported [18]. Some of these strains such as *Klebsiella* spp. are responsible of causing pneumonia [36–40].

### 4.1. Strength and Limitations of the Study

This study is conducted in one season and may not reflect the actual prevalence of pneumonia among the study participants. Recall bias and lack of laboratory confirmation of the pneumonia symptoms are the other limitations of the study. The use of large sample size is an advantage in this study.

### 4.2. Implications for Policymakers

The evidence from this finding calls for policymakers to play a role in reducing childhood mortality and morbidity imposed by pneumonia through controlling indoor and outdoor air pollutions, ensuring adequate ventilation, applying mechanisms in vector control, and improving environmental cleanliness and safety which are also endorsed by the global policies for pneumonia control.

### 4.3. Implication for Future Research

Although this study is no longer sufficient to confirm the association between childhood pneumonia and malnutrition history, sufficient breastfeeding, immunization status, low birth weight, and hereditary diseases of the respiratory tract, there are evidences showing that these variables have a share on the global burden of pneumonia morbidity and mortality. Thus, further high-quality study is needed in this area aided by laboratory tests like a lung function test to better produce a high-level evidence.

## 5. Conclusions

A high prevalence of community-acquired pneumonia was found among under-five children in the study area. The presence of unpaved road within 100 m of the house, living within 100 m of heavy traffic, the habit of not opening doors while cooking, cockroach infestation, and new carpet in the house were risk factors. Town planning and zoning, ventilation, and vector control are important interventions to reduce under-five pneumonia that arises and is aggravated from environmental exposure.

## Figures and Tables

**Table 1:** Sociodemographic characteristics of the study participants in Gondar City, Northwest Ethiopia, 2019.

Variables	Frequency	Percent (%)
Sex of child		
Male	431	54.4
Female	361	45.6
Age of child		
1 year	204	25.8
2 years	213	26.9
3 years	181	22.9
≥4 years	194	24.5
Age of mothers		
<25 years	157	19.8
25-27 years	198	25.0
28-31 years	217	27.4
≥32 years	220	27.8
Average monthly income		
200-1999 ETB	153	19.3
2000-3549 ETB	253	31.9
3550-4999 ETB	174	22.0
≥5000 ETB	212	26.8
Religion		
Orthodox	610	77.0
Muslim	140	17.7
Others^∗^	42	5.3
Current marital status of women		
Single	40	5.1
Married	684	86.4
Divorced	35	4.4
Widowed	8	1.0
Separated	25	3.2
Educational status of mothers/guardians		
Unable to read and write	135	17.0
Read and write	90	11.4
Primary	100	12.6
Secondary	253	31.9
Graduate from vocational	30	3.8
Diploma and above	184	23.3
Occupational status of mothers/guardians		
Housewife	541	68.3
Farmer	4	0.5
Student	8	1.0
Private employee	52	6.6
Government employee	142	17.9
Merchant	35	4.4
Others^∗∗^	10	1.3

Others^∗^ = Protestant, Jewish, or Catholic. Others^∗∗^ = daily laborer, driver, or priest.

**Table 2:** Factors associated with childhood pneumonia in Gondar City, Northwest Ethiopia (*n* = 792).

Variables	Pneumonia		COR (95% CI)	AOR (95% CI)
	Yes (%)	No (%)		
Child exposure to pets				
Yes	9 (23.7)	29 (76.3)	2.38 (1.09, 5.19)	11.45 (0.62, 3.38)
No	87 (11.5)	667 (88.5)	1	1
Presence of unpaved road within 100 m of the house				
Yes	43 (22.2)	151 (77.8)	2.93 (1.88, 4.55)	2.27 (1.41, 3.66)^∗∗^
No	53 (8.9)	545 (91.1)	1	
Living within 100 m of heavy traffic				
Yes	34 (21.0)	128 (79.0)	2.43 (1.54, 3.86)	1.94 (1.19, 3.16)^∗∗^
No	62 (9.8)	568 (90.2)	1	1
Opening door during cooking				
Yes	33 (9.3)	320 (90.7)	1	1
No	63 (14.4)	376 (85.6)	1.63 (1.04, 2.54)	1.62 (1.01, 2.62)^∗^
Presence of damp stain				
Yes	13 (20.6)	50 (79.4)	2.02 (1.06, 3.88)	1.63 (0.79, 3.36)
No	83 (11.4)	646 (88.6)	1	1
Presence of new carpet in the house				
Yes	23 (20.9)	87 (79.1)	2.21 (1.31, 3.71)	1.75 (1.01, 3.03)^∗^
No	73 (10.7)	609 (89.3)	1	1
Cockroach infestation				
Yes	58 (15.3)	321 (84.7)	1.78 (1.15, 2.76)	1.98 (1.25, 3.14)^∗∗^
No	38 (9.2)	375 (90.8)	1	1
Maternal cough				
Yes	19 (20.4)	74 (79.6)	2.07 (1.19, 3.62)	1.70 (0.93, 3.10)
No	77 (11.0)	622 (89.0)	1	1
Maternal wheezing				
Yes	11 (22.4)	38 (77.6)	2.24 (1.10, 4.55)	1.83 (0.86, 3.88)
No	85 (11.4)	658 (88.6)	1	1

^∗^ *p* < 0.05, ^∗∗^*p* ≤ 0.001, Hosmer and Lemeshow goodness of fit (*p* = 0.616).

## Data Availability

The dataset analyzed during the current study is available from the corresponding author on reasonable request.

## Abbreviations

AOR: Adjusted odds ratio
CI: Confidence interval
EDHS: Ethiopian Demographic Health Survey
ETB: Ethiopian birr
LMIC: Low- and middle-income countries
OR: Odds ratio
SPSS: Statistical Package for the Social Sciences
WHO: World Health Organization.
